# Supplementary material for: A high-resolution mRNA expression time course of embryonic development in zebrafish
Source: eLife. 2017 Nov 16;6:e30860. doi: 10.7554/eLife.30860 (PMC5690287; doi:10.7554/eLife.30860)
Supplement: Supplementary file 6. [file elife-30860-supp6.zip › biolayout-clusters-files/Cluster165-genes.html]

Cluster165


# Cluster165: Genes

| Ensembl ID | Gene Name | Chr | Start | End | Biotype |
| --- | --- | --- | --- | --- | --- |
| ENSDARG00000037122 | TENM2 (1 of many).1 | 14 | 28860295 | 29419805 | protein\_coding |
| ENSDARG00000098140 | ankrd13d | 14 | 21485402 | 21519461 | protein\_coding |
| ENSDARG00000087993 | bada | 21 | 27152792 | 27154509 | protein\_coding |
| ENSDARG00000037478 | cdh8 | 7 | 42794171 | 42972949 | protein\_coding |
| ENSDARG00000098652 | pcdh11 | 14 | 26975638 | 26999424 | protein\_coding |
| ENSDARG00000044183 | prkab1a | 10 | 17979007 | 17988729 | protein\_coding |
| ENSDARG00000052011 | rrad | 7 | 44620963 | 44623929 | protein\_coding |
| ENSDARG00000086104 | slco5a2 | 2 | 42533043 | 42566484 | protein\_coding |
